# Aqua­(imino­diacetato-κ^3^ *O*,*N*,*O*′)(1,10-phenanthroline-κ^2^ *N*,*N*′)zinc(II) sesquihydrate

**DOI:** 10.1107/S1600536808042141

**Published:** 2008-12-17

**Authors:** Hwa Loong Ng, Chew Hee Ng, Seik Weng Ng

**Affiliations:** a Faculty of Engineering & Science, Universiti Tunku Abdul Rahman, Jalan Genting Kelang, 53100 Kuala Lumpur, Malaysia; b Department of Chemistry, University of Malaya, 50603 Kuala Lumpur, Malaysia

## Abstract

The imino­diacetate dianion in the title compound, [Zn(C_4_H_5_NO_4_)(C_12_H_8_N_2_)(H_2_O)]·1.5H_2_O, chelates to the Zn^II^ center with its N and two O atoms. The metal atom is also chelated by the *N*-heterocycle and coordinated by one water molecule, leading to a distorted octahedral environment. The dianion, and coordinated and uncoordinated water mol­ecules inter­act through O—H⋯O hydrogen bonds, generating a three-dimensional network. One of the two uncoordinated water mol­ecules has half-site occupancy. The crystal studied was a non-merohedral twin with a 15% twin component.

## Related literature

For the structure of zinc bis­[imino­diacetate­(1-)] tetra­hydrate, see: Sinkha *et al.* (1975 ▶). For the dihydrated adenine adduct of zinc imino­diacetate, see: Morel *et al.* (2003 ▶). For the use of *PLATON* to separate twin fractions from diffraction data, see: Spek (2003 ▶).
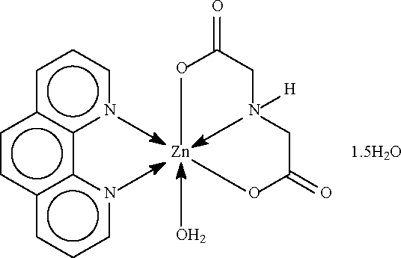


## Experimental

### 

#### Crystal data

[Zn(C_4_H_5_NO_4_)(C_12_H_8_N_2_)(H_2_O)]·1.5H_2_O
*M* *_r_* = 421.70
Triclinic, 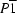

*a* = 6.5989 (1) Å
*b* = 10.6440 (1) Å
*c* = 11.5456 (2) Å
α = 95.156 (1)°
β = 91.845 (1)°
γ = 92.190 (1)°
*V* = 806.56 (2) Å^3^
*Z* = 2
Mo *K*α radiation
μ = 1.57 mm^−1^
*T* = 100 (2) K
0.35 × 0.25 × 0.15 mm

#### Data collection

Bruker SMART APEX diffractometer
Absorption correction: multi-scan (*SADABS*; Sheldrick, 1996 ▶) *T*_min_ = 0.610, *T*_max_ = 0.799
7242 measured reflections
3640 independent reflections
3455 reflections with *I* > 2σ(*I*)
*R*_int_ = 0.025

#### Refinement

*R*[*F* ^2^ > 2σ(*F* ^2^)] = 0.048
*wR*(*F* ^2^) = 0.146
*S* = 1.22
3640 reflections
233 parameters
72 restraints
H-atom parameters constrained
Δρ_max_ = 1.02 e Å^−3^
Δρ_min_ = −0.88 e Å^−3^

### 

Data collection: *APEX2* (Bruker, 2007 ▶); cell refinement: *SAINT* (Bruker, 2007 ▶); data reduction: *SAINT*; program(s) used to solve structure: *SHELXS97* (Sheldrick, 2008 ▶); program(s) used to refine structure: *SHELXL97* (Sheldrick, 2008 ▶); molecular graphics: *X-SEED* (Barbour, 2001 ▶); software used to prepare material for publication: *publCIF* (Westrip, 2009 ▶).

## Supplementary Material

Crystal structure: contains datablocks global, I. DOI: 10.1107/S1600536808042141/xu2469sup1.cif

Structure factors: contains datablocks I. DOI: 10.1107/S1600536808042141/xu2469Isup2.hkl

Additional supplementary materials:  crystallographic information; 3D view; checkCIF report

## Figures and Tables

**Table 1:** Hydrogen-bond geometry (Å, °)

*D*—H⋯*A*	*D*—H	H⋯*A*	*D*⋯*A*	*D*—H⋯*A*
O1w—H11⋯O1^i^	0.84	2.17	2.801 (4)	132
O1w—H12⋯O3^ii^	0.84	1.92	2.757 (4)	172
O2w—H21⋯O2^i^	0.84	1.98	2.815 (5)	177
O2w—H22⋯O4^iii^	0.84	1.92	2.756 (5)	177
O3w—H31⋯O2w	0.84	1.94	2.780 (9)	174

Symmetry codes: (i) 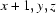; (ii) 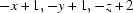; (iii) 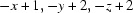.

**Fig. 1.**
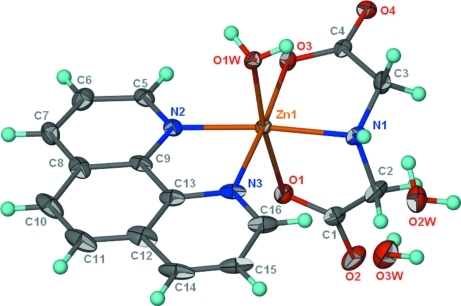
Thermal ellipsoid plot (Barbour, 2001) of Zn(H2O)(C4H5NO4)(C12H8N2).1.5H2O at the 70% probability level. Hydrogen atoms are drawn as spheres of arbitrary radius. The O3w water molecule, which lies on a general position, has 0.5 occupancy.

## supplementary crystallographic information

### Experimental

An methanol solution of zinc(II) nitrate hexahydrate (0.30 g, 1 mmol) and 1,10-phenanthroline (0.20 g, 1 mmol) was mixed with an aqueous solution of iminodiacetic acid (0.14 g, 1 mmol) and sodium hydroxide (0.08 g, 2 mmol). The mixture was briefly heated. The cool solution yielded a white solid. This was recrystallized from a water-methanol mixture to give colorless crystals.

### Refinement

Carbon- and nitrogen-bound hydrogen atoms were placed at calculated positions (C–H 0.95–0.98 Å, N–H 0.88 Å) and were treated as riding on their parent atoms, with *U*(H) set to 1.2 times *U*_eq_(*C *or *N*). The water H-atoms were placed in chemically-sensible positions on the basis of hydrogen bonding, but were not refined; their temperature factors were tied by a factor of 1.5.

For the three phenanthroline groups, the central six-membered ring was refined as a rigid hexagon of 1.39 Å sides. The temperature factors of the carbon atoms of this fused-ring system were restrained to be nearly isotropic.

The O3*w* atom gave a large temperature factor when allowed to refined at full occupancy. The occupancy could be refined, and this refined to nearly 0.5. As such, the occupancy was then fixed as exactly 0.5. This water molecule was within hydrogen bonding distance of only one other acceptor atom.

The structure is a non-merohedral twin. *PLATON* (Spek, 2003) was used to de-twin the structure. The twin component refined to 15%; the inclusion of the twin law lowered the *R* index from 6.4%. More importantly, it improved the weighting scheme. The final difference Fourier map was now diffuse, with the largest peak of slighly over 1 *e* Å^-3^ in the vicinity of C12.

### Crystal data

**Table:**

[Zn(C_4_H_5_NO_4_)(C_12_H_8_N_2_)(H_2_O)]·1.5H_2_O	*Z* = 2
*M**_r_* = 421.70	*F*(000) = 434
Triclinic, *P*1	*D*_x_ = 1.736 Mg m^−^^3^
Hall symbol: -P 1	Mo *K*α radiation, λ = 0.71073 Å
*a* = 6.5989 (1) Å	Cell parameters from 6223 reflections
*b* = 10.6440 (1) Å	θ = 2.5–28.3°
*c* = 11.5456 (2) Å	µ = 1.57 mm^−^^1^
α = 95.156 (1)°	*T* = 100 K
β = 91.845 (1)°	Block, colorless
γ = 92.190 (1)°	0.35 × 0.25 × 0.15 mm
*V* = 806.56 (2) Å^3^	

### Data collection

**Table:**

Bruker SMART APEX diffractometer	3640 independent reflections
Radiation source: fine-focus sealed tube	3455 reflections with *I* > 2σ(*I*)
graphite	*R*_int_ = 0.025
ω scans	θ_max_ = 27.5°, θ_min_ = 1.8°
Absorption correction: multi-scan (*SADABS*; Sheldrick, 1996)	*h* = −8→8
*T*_min_ = 0.610, *T*_max_ = 0.799	*k* = −13→13
7242 measured reflections	*l* = −14→14

### Refinement

**Table:**

Refinement on *F*^2^	Primary atom site location: structure-invariant direct methods
Least-squares matrix: full	Secondary atom site location: difference Fourier map
*R*[*F*^2^ > 2σ(*F*^2^)] = 0.048	Hydrogen site location: inferred from neighbouring sites
*wR*(*F*^2^) = 0.146	H-atom parameters constrained
*S* = 1.22	*w* = 1/[σ^2^(*F*_o_^2^) + (0.0241*P*)^2^ + 4.8628*P*] where *P* = (*F*_o_^2^ + 2*F*_c_^2^)/3
3640 reflections	(Δ/σ)_max_ = 0.001
233 parameters	Δρ_max_ = 1.01 e Å^−^^3^
72 restraints	Δρ_min_ = −0.88 e Å^−^^3^

### Fractional atomic coordinates and isotropic or equivalent isotropic displacement parameters (Å^2^)

**Table:**

	*x*	*y*	*z*	*U*_iso_*/*U*_eq_	Occ. (<1)
Zn1	0.30260 (7)	0.63033 (5)	0.84683 (4)	0.01073 (15)	
O1	−0.0016 (5)	0.7036 (3)	0.8533 (3)	0.0154 (6)	
O2	−0.1646 (5)	0.8841 (3)	0.8519 (4)	0.0242 (8)	
O3	0.3125 (5)	0.6228 (3)	1.0256 (3)	0.0137 (6)	
O4	0.3975 (5)	0.7409 (3)	1.1911 (3)	0.0168 (7)	
O1W	0.6167 (5)	0.5782 (3)	0.8460 (3)	0.0137 (6)	
H11	0.6902	0.6376	0.8795	0.021*	
H12	0.6294	0.5131	0.8812	0.021*	
O2W	0.5864 (6)	1.0151 (3)	0.7055 (3)	0.0270 (8)	
H21	0.6601	0.9737	0.7475	0.040*	
H22	0.5886	1.0902	0.7349	0.040*	
O3W	0.7199 (12)	1.0113 (7)	0.4793 (7)	0.0287 (16)	0.50
H31	0.6705	1.0115	0.5455	0.043*	0.50
H32	0.7870	1.0790	0.4740	0.043*	0.50
N1	0.3717 (6)	0.8255 (3)	0.8921 (3)	0.0155 (8)	
H1	0.4735	0.8496	0.8507	0.019*	
N2	0.1966 (5)	0.4443 (4)	0.7994 (3)	0.0142 (7)	
N3	0.3044 (5)	0.6254 (4)	0.6611 (3)	0.0157 (7)	
C1	−0.0062 (7)	0.8228 (4)	0.8568 (4)	0.0153 (8)	
C2	0.1934 (7)	0.8985 (4)	0.8650 (5)	0.0207 (10)	
H2A	0.2137	0.9349	0.7901	0.025*	
H2B	0.1849	0.9696	0.9259	0.025*	
C3	0.4372 (7)	0.8367 (4)	1.0155 (4)	0.0178 (9)	
H3A	0.3811	0.9141	1.0538	0.021*	
H3B	0.5869	0.8478	1.0206	0.021*	
C4	0.3754 (6)	0.7252 (4)	1.0842 (4)	0.0127 (8)	
C5	0.1390 (6)	0.3577 (4)	0.8671 (4)	0.0149 (8)	
H5	0.1355	0.3802	0.9485	0.018*	
C6	0.0828 (7)	0.2336 (5)	0.8242 (5)	0.0200 (9)	
H6	0.0424	0.1735	0.8760	0.024*	
C7	0.0864 (7)	0.1993 (5)	0.7068 (4)	0.0198 (9)	
H7	0.0504	0.1151	0.6768	0.024*	
C9	0.1991 (4)	0.4127 (3)	0.67929 (18)	0.0146 (8)	
C8	0.1450 (5)	0.2916 (2)	0.6299 (2)	0.0186 (9)	
C10	0.1399 (5)	0.2654 (2)	0.5097 (3)	0.0265 (11)	
H10	0.1029	0.1827	0.4759	0.032*	
C11	0.1889 (5)	0.3603 (3)	0.43886 (18)	0.0271 (11)	
H11A	0.1854	0.3423	0.3567	0.032*	
C12	0.2430 (5)	0.4813 (3)	0.4883 (2)	0.0213 (10)	
C13	0.2481 (4)	0.5075 (2)	0.6085 (2)	0.0168 (9)	
C14	0.2936 (7)	0.5828 (6)	0.4185 (4)	0.0254 (11)	
H14	0.2912	0.5683	0.3360	0.030*	
C15	0.3446 (7)	0.6986 (5)	0.4710 (4)	0.0220 (10)	
H15	0.3765	0.7668	0.4262	0.026*	
C16	0.3497 (7)	0.7169 (5)	0.5941 (4)	0.0209 (10)	
H16	0.3874	0.7985	0.6304	0.025*	

### Atomic displacement parameters (Å^2^)

**Table:**

	*U*^11^	*U*^22^	*U*^33^	*U*^12^	*U*^13^	*U*^23^
Zn1	0.0119 (2)	0.0110 (2)	0.0096 (2)	0.00101 (17)	0.00101 (17)	0.00212 (17)
O1	0.0118 (14)	0.0139 (14)	0.0205 (16)	−0.0007 (11)	0.0005 (12)	0.0029 (12)
O2	0.0160 (16)	0.0179 (16)	0.040 (2)	0.0051 (13)	0.0028 (15)	0.0070 (15)
O3	0.0180 (15)	0.0111 (14)	0.0121 (15)	0.0005 (12)	0.0027 (12)	0.0010 (11)
O4	0.0199 (16)	0.0168 (15)	0.0137 (15)	0.0026 (12)	−0.0020 (12)	0.0013 (12)
O1W	0.0139 (14)	0.0114 (14)	0.0162 (15)	−0.0003 (11)	−0.0003 (12)	0.0035 (12)
O2W	0.036 (2)	0.0172 (17)	0.0267 (19)	0.0008 (15)	−0.0018 (16)	−0.0029 (14)
O3W	0.038 (4)	0.023 (4)	0.026 (4)	0.005 (3)	0.006 (3)	0.003 (3)
N1	0.0130 (18)	0.0134 (17)	0.021 (2)	0.0021 (14)	0.0008 (14)	0.0051 (14)
N2	0.0089 (16)	0.0159 (18)	0.0175 (19)	0.0032 (13)	−0.0017 (14)	−0.0013 (14)
N3	0.0078 (16)	0.027 (2)	0.0135 (18)	0.0044 (14)	0.0017 (13)	0.0069 (15)
C1	0.014 (2)	0.017 (2)	0.016 (2)	0.0014 (16)	0.0028 (16)	0.0052 (17)
C2	0.013 (2)	0.014 (2)	0.037 (3)	0.0018 (16)	0.0014 (19)	0.0100 (19)
C3	0.023 (2)	0.0123 (19)	0.018 (2)	−0.0015 (17)	0.0011 (18)	−0.0002 (16)
C4	0.0091 (18)	0.0126 (19)	0.017 (2)	0.0027 (15)	0.0023 (15)	0.0016 (16)
C5	0.0088 (18)	0.019 (2)	0.016 (2)	0.0008 (15)	−0.0006 (15)	−0.0006 (16)
C6	0.016 (2)	0.018 (2)	0.027 (2)	0.0023 (17)	0.0023 (18)	0.0038 (18)
C7	0.014 (2)	0.019 (2)	0.025 (2)	0.0023 (17)	−0.0019 (18)	−0.0068 (18)
C9	0.0073 (18)	0.024 (2)	0.013 (2)	0.0044 (16)	0.0002 (15)	0.0001 (17)
C8	0.0083 (19)	0.024 (2)	0.023 (2)	0.0015 (16)	0.0000 (16)	−0.0039 (18)
C10	0.011 (2)	0.042 (3)	0.024 (2)	0.004 (2)	−0.0026 (18)	−0.012 (2)
C11	0.013 (2)	0.050 (3)	0.016 (2)	0.005 (2)	−0.0006 (17)	−0.006 (2)
C12	0.0075 (18)	0.041 (3)	0.016 (2)	0.0062 (18)	0.0046 (16)	0.0011 (19)
C13	0.0101 (19)	0.026 (2)	0.015 (2)	0.0059 (17)	0.0009 (15)	0.0034 (17)
C14	0.014 (2)	0.053 (3)	0.010 (2)	0.010 (2)	0.0018 (17)	0.008 (2)
C15	0.012 (2)	0.042 (3)	0.016 (2)	0.0095 (19)	0.0052 (16)	0.015 (2)
C16	0.013 (2)	0.033 (3)	0.018 (2)	0.0052 (18)	0.0019 (17)	0.0109 (19)

### Geometric parameters (Å, °)

**Table:**

Zn1—O3	2.072 (3)	C2—H2B	0.9900
Zn1—N2	2.093 (4)	C3—C4	1.535 (6)
Zn1—N1	2.124 (4)	C3—H3A	0.9900
Zn1—N3	2.141 (4)	C3—H3B	0.9900
Zn1—O1W	2.166 (3)	C5—C6	1.401 (6)
Zn1—O1	2.182 (3)	C5—H5	0.9500
O1—C1	1.267 (5)	C6—C7	1.373 (7)
O2—C1	1.255 (5)	C6—H6	0.9500
O3—C4	1.278 (5)	C7—C8	1.433 (6)
O4—C4	1.233 (6)	C7—H7	0.9500
O1W—H11	0.8399	C9—C8	1.3900
O1W—H12	0.8400	C9—C13	1.3900
O2W—H21	0.8400	C8—C10	1.3900
O2W—H22	0.8400	C10—C11	1.3900
O3W—H31	0.8401	C10—H10	0.9500
O3W—H32	0.8399	C11—C12	1.3900
N1—C3	1.469 (6)	C11—H11A	0.9500
N1—C2	1.475 (6)	C12—C13	1.3900
N1—H1	0.8800	C12—C14	1.440 (6)
N2—C5	1.315 (6)	C14—C15	1.350 (8)
N2—C9	1.398 (4)	C14—H14	0.9500
N3—C16	1.329 (6)	C15—C16	1.415 (7)
N3—C13	1.376 (5)	C15—H15	0.9500
C1—C2	1.513 (6)	C16—H16	0.9500
C2—H2A	0.9900		
			
O3—Zn1—N2	98.01 (14)	N1—C3—H3A	108.3
O3—Zn1—N1	83.15 (14)	C4—C3—H3A	108.3
N2—Zn1—N1	172.78 (14)	N1—C3—H3B	108.3
O3—Zn1—N3	175.75 (14)	C4—C3—H3B	108.3
N2—Zn1—N3	79.24 (16)	H3A—C3—H3B	107.4
N1—Zn1—N3	99.99 (16)	O4—C4—O3	125.7 (4)
O3—Zn1—O1W	88.31 (12)	O4—C4—C3	117.1 (4)
N2—Zn1—O1W	92.50 (13)	O3—C4—C3	117.2 (4)
N1—Zn1—O1W	94.66 (13)	N2—C5—C6	122.7 (4)
N3—Zn1—O1W	88.57 (13)	N2—C5—H5	118.6
O3—Zn1—O1	90.74 (12)	C6—C5—H5	118.6
N2—Zn1—O1	93.69 (13)	C7—C6—C5	119.5 (5)
N1—Zn1—O1	79.15 (13)	C7—C6—H6	120.3
N3—Zn1—O1	92.67 (13)	C5—C6—H6	120.3
O1W—Zn1—O1	173.81 (12)	C6—C7—C8	119.5 (4)
C1—O1—Zn1	114.4 (3)	C6—C7—H7	120.3
C4—O3—Zn1	114.8 (3)	C8—C7—H7	120.3
Zn1—O1W—H11	109.5	C8—C9—C13	120.0
Zn1—O1W—H12	109.4	C8—C9—N2	121.7 (2)
H11—O1W—H12	109.5	C13—C9—N2	118.2 (2)
H21—O2W—H22	108.3	C10—C8—C9	120.0
H31—O3W—H32	110.0	C10—C8—C7	122.4 (3)
C3—N1—C2	114.7 (4)	C9—C8—C7	117.5 (3)
C3—N1—Zn1	105.8 (3)	C8—C10—C11	120.0
C2—N1—Zn1	109.4 (3)	C8—C10—H10	120.0
C3—N1—H1	108.9	C11—C10—H10	120.0
C2—N1—H1	108.9	C12—C11—C10	120.0
Zn1—N1—H1	108.9	C12—C11—H11A	120.0
C5—N2—C9	119.1 (4)	C10—C11—H11A	120.0
C5—N2—Zn1	128.5 (3)	C13—C12—C11	120.0
C9—N2—Zn1	112.3 (3)	C13—C12—C14	118.0 (3)
C16—N3—C13	118.5 (4)	C11—C12—C14	122.0 (3)
C16—N3—Zn1	129.7 (4)	N3—C13—C12	121.9 (2)
C13—N3—Zn1	111.8 (2)	N3—C13—C9	118.1 (2)
O2—C1—O1	125.1 (4)	C12—C13—C9	120.0
O2—C1—C2	116.7 (4)	C15—C14—C12	119.5 (4)
O1—C1—C2	118.3 (4)	C15—C14—H14	120.2
N1—C2—C1	114.4 (4)	C12—C14—H14	120.2
N1—C2—H2A	108.7	C14—C15—C16	119.0 (5)
C1—C2—H2A	108.7	C14—C15—H15	120.5
N1—C2—H2B	108.7	C16—C15—H15	120.5
C1—C2—H2B	108.7	N3—C16—C15	123.0 (5)
H2A—C2—H2B	107.6	N3—C16—H16	118.5
N1—C3—C4	115.9 (4)	C15—C16—H16	118.5
			
O3—Zn1—O1—C1	95.8 (3)	Zn1—O3—C4—C3	−1.0 (5)
N2—Zn1—O1—C1	−166.1 (3)	N1—C3—C4—O4	−167.9 (4)
N1—Zn1—O1—C1	12.9 (3)	N1—C3—C4—O3	14.5 (6)
N3—Zn1—O1—C1	−86.8 (3)	C9—N2—C5—C6	1.0 (6)
N2—Zn1—O3—C4	179.4 (3)	Zn1—N2—C5—C6	−175.9 (3)
N1—Zn1—O3—C4	−7.8 (3)	N2—C5—C6—C7	−0.2 (7)
O1W—Zn1—O3—C4	87.1 (3)	C5—C6—C7—C8	−0.9 (7)
O1—Zn1—O3—C4	−86.8 (3)	C5—N2—C9—C8	−0.8 (5)
O3—Zn1—N1—C3	14.2 (3)	Zn1—N2—C9—C8	176.55 (15)
N3—Zn1—N1—C3	−162.9 (3)	C5—N2—C9—C13	176.0 (3)
O1W—Zn1—N1—C3	−73.6 (3)	Zn1—N2—C9—C13	−6.6 (3)
O1—Zn1—N1—C3	106.2 (3)	C13—C9—C8—C10	0.0
O3—Zn1—N1—C2	−110.0 (3)	N2—C9—C8—C10	176.8 (3)
N3—Zn1—N1—C2	72.9 (3)	C13—C9—C8—C7	−177.0 (3)
O1W—Zn1—N1—C2	162.3 (3)	N2—C9—C8—C7	−0.2 (4)
O1—Zn1—N1—C2	−17.9 (3)	C6—C7—C8—C10	−175.9 (3)
O3—Zn1—N2—C5	5.3 (4)	C6—C7—C8—C9	1.0 (5)
N3—Zn1—N2—C5	−178.0 (4)	C9—C8—C10—C11	0.0
O1W—Zn1—N2—C5	94.0 (4)	C7—C8—C10—C11	176.9 (3)
O1—Zn1—N2—C5	−85.9 (4)	C8—C10—C11—C12	0.0
O3—Zn1—N2—C9	−171.7 (2)	C10—C11—C12—C13	0.0
N3—Zn1—N2—C9	5.0 (2)	C10—C11—C12—C14	−179.6 (3)
O1W—Zn1—N2—C9	−83.1 (3)	C16—N3—C13—C12	1.8 (5)
O1—Zn1—N2—C9	97.0 (3)	Zn1—N3—C13—C12	−178.48 (15)
N2—Zn1—N3—C16	176.8 (4)	C16—N3—C13—C9	−179.4 (3)
N1—Zn1—N3—C16	4.1 (4)	Zn1—N3—C13—C9	0.3 (3)
O1W—Zn1—N3—C16	−90.4 (4)	C11—C12—C13—N3	178.8 (3)
O1—Zn1—N3—C16	83.5 (4)	C14—C12—C13—N3	−1.6 (4)
N2—Zn1—N3—C13	−2.9 (2)	C11—C12—C13—C9	0.0
N1—Zn1—N3—C13	−175.6 (2)	C14—C12—C13—C9	179.6 (3)
O1W—Zn1—N3—C13	89.9 (3)	C8—C9—C13—N3	−178.8 (3)
O1—Zn1—N3—C13	−96.2 (3)	N2—C9—C13—N3	4.2 (3)
Zn1—O1—C1—O2	174.2 (4)	C8—C9—C13—C12	0.0
Zn1—O1—C1—C2	−4.4 (5)	N2—C9—C13—C12	−176.9 (3)
C3—N1—C2—C1	−97.2 (5)	C13—C12—C14—C15	0.2 (5)
Zn1—N1—C2—C1	21.6 (5)	C11—C12—C14—C15	179.8 (3)
O2—C1—C2—N1	169.4 (4)	C12—C14—C15—C16	0.9 (7)
O1—C1—C2—N1	−11.9 (7)	C13—N3—C16—C15	−0.6 (6)
C2—N1—C3—C4	101.9 (4)	Zn1—N3—C16—C15	179.8 (3)
Zn1—N1—C3—C4	−18.9 (4)	C14—C15—C16—N3	−0.8 (7)
Zn1—O3—C4—O4	−178.3 (3)		

### Hydrogen-bond geometry (Å, °)

**Table:**

*D*—H···*A*	*D*—H	H···*A*	*D*···*A*	*D*—H···*A*
N1—H1···O2w	0.88	2.64	3.388 (5)	143
O1w—H11···O1^i^	0.84	2.17	2.801 (4)	132
O1w—H12···O3^ii^	0.84	1.92	2.757 (4)	172
O2w—H21···O2^i^	0.84	1.98	2.815 (5)	177
O2w—H22···O4^iii^	0.84	1.92	2.756 (5)	177
O3w—H31···O2w	0.84	1.94	2.780 (9)	174

Symmetry codes: (i) *x*+1, *y*, *z*; (ii) −*x*+1, −*y*+1, −*z*+2; (iii) −*x*+1, −*y*+2, −*z*+2.

## References

[bb1] Barbour, L. J. (2001). *J. Supramol. Chem.* **1**, 189–191.

[bb2] Bruker (2007). *APEX2* and *SAINT* Bruker AXS Inc., Madison, Wisconsin, USA.

[bb3] Morel, C., Choquesillo-Lazarte, D., Alarcón-Payer, C., González-Pérez, J. M., Castiñeiras, A. & Niclós-Gutiérrez, J. (2003). *Inorg. Chem. Commun.* **11**, 1354–1357.

[bb4] Sheldrick, G. M. (1996). *SADABS* University of Göttingen, Germany.

[bb5] Sheldrick, G. M. (2008). *Acta Cryst.* A**64**, 112–122. 10.1107/S0108767307043930 18156677

[bb6] Sinkha, U.-Ch., Kramarenko, F. G., Polynova, T. N., Porai-Koshits, M. A. & Mitrofanova, N. D. (1975). *Zh. Strukt. Khim.* **16**, 144–145.

[bb7] Spek, A. L. (2003). *J. Appl. Cryst.* **36**, 7–13.

[bb8] Westrip, S. P. (2009). *publCIF* In preparation.

